# The novel coronavirus outbreak: what can be learned from China in public reporting?

**DOI:** 10.1186/s41256-020-00140-9

**Published:** 2020-03-09

**Authors:** Hao Li, Xinguang Chen, Hao Huang

**Affiliations:** 1 Global Health Institute, Wuhan University, Wuhan, 430072 China; 2 Department of Epidemiology, University of Florida, Gainesville, 32610 USA; 3 School of Political Science and Public Administration, Wuhan University, Wuhan, 430072 China

**Keywords:** Coronavirus outbreak, COVID-19, 2019-nCoV, SARS-CoV-2, Epidemic, Pandemic, Public reporting, Indicators, Surveillance, China

## Abstract

The new coronavirus outbreak gets everyone’s attention. China’s national actions against the outbreak have contributed great contributions to the world. China has been learning from practice for better reporting and is fast to adapt itself. In this article we discuss China’s practice in public reporting and its implications to global health. Confirmed cases, dynamic suspected cases, recovered cases, and deaths have been reported both in accumulative numbers and their daily updates. Some ratio indictors reporting (fatality rate, recovery rate, etc.), trend reporting, and global surveillance have been applied as well. Some improvements can still be made. It is necessary to further explore the influential factors behind the indicators for interventions. Recommendations are made to the World Health Organization and other countries for better public reporting and surveillance.

## Background

The storm-like outbreak of the novel coronavirus epidemic, named as COVID-19, or 2019-nCoV, or SARS-CoV-2, gets everyone’s attention [1]. Since the first case was reported in Wuhan (China) on December 8, 2019 [2], until February 27, 2020, a total of 78,497 cases have been confirmed, 32,495 cases recovered and 2744 cases died in China [3]. Up to date, the outbreak has already been spread to 37 countries worldwide and a total of 2918 cases have been confirmed, 287 recovered, and 43 cases died [4, 5]. Had it not for the whole society effort led by the Chinese government, the situation would be much worse in China and around the world. For better global actions, there is a urgent need for public reporting among Nations and further global surveillance.

Moreover, as there lacks effective drugs and people are easy to be infected via contact, droplets, etc., worries and panic have been aroused in the public in China and other countries. Scientifically framed epidemic information about the outbreak can help people understand the developing situation, encourage the whole society to work together against the epidemic, and help prevent data from deliberate misinterpretation. In theory, all relevant information can be reported to support decision making, while such approach takes a lot of time and make it impossible to do so. Therefore, for timely public reporting, selecting the most representive indicators are of most importance. China is the first country to fight the outbreak and have accumulated lots of exprience. Its experience to report the indictors information to the public on daily basis is very useful not only for the World Health Organization (WHO) to strengthen global surveillance, but also for other countries to improve their public reporting.

## Current reporting progress in China

Since the outbreak, reporting to the public has been developing from exploration all the time. From one side, governments of all levels announce new updates each day and reflect all indicators information via the Chinese Center for Disease Control and Prevention (CDC) [3]; on the other side, many domestic search engines, portal websites, etc. have developed their own surveillance systems respectively and update information each day based on the newest data officially from governments of all levels, which include case number indicators, trend monitoring, news from various sources (such as webpages, TV, newspapers, blogs, broadcasts, ect.) [6, 7]. Each of them have their own features and have attracted huge number of visits each day for concerns. Moreover, the reporting not only include information updates about China, but also the world.

Further analysis of the government reports indicate that indicators such as confirmed cases, suspected cases, recovered cases, and total number of deaths are all reported in total numbers along with their daily updates respectively based on provincial and city levels. These data together with the updates from the WHO have provided good basis for visual reporting at city, provincial, national, and international levels. Ratio analyses such as case fatality rate, recovery rate, etc. are applied to reflect the whole progress of various effort and interventions. These rates are further classified as Hubei, outside Hubei, whole China, etc. for comparison in curves. It should be noted that there are lots of influential factors affecting the fatality and recovery rates [8], for example, the fatility rate in Hubei is much higher than other provinces (4.03% vs 0.80%, by Feburuary 27, 2020), which can be partly explained that, even though Hubei is abundant of health resources, but still lacks enough medical resources to provide timely and effective treatments to the hug number of patients compared with much less patients in other Chinese provinces.

Incidence and case mortality rate can both be meaningful to be reported. Incidence indicates the infectivity of the virus over a time period, and case mortality rate can reflect the virulence of the new coronavirus. Both are yet to be reflected in reporting. It will be much helpful to report up to date incidence and case mortality rate for daily surveillance, and a trend curve can be depicted, which can indicate the cumulative risks and remind people to end the outbreak as soon as possible.

## Implications for global health

Based on China’s advantage in information communications technology (ICT), the entreprenurship of technology enterprises, etc., China has developed a relatively complete online reporting system to the public in a very short time. However, with the outbreak developing into a global pandemic, many countries are still underestimating the impact of the outbreak. The following aspects can be highlited for public reporting, surveillance and actions:

1. The number of deaths can further be classified by gender and age. In this way the information will be much helpful to further identify susceptible populations.
2. According to China’s practice, the number of confirmed cases is better to include only laboratory confirmed cases, and all clinically diagnostic cases should all be included into suspected cases before they are confirmed by laboratory tests. However, this creates the difficulty for countries lacking test capabilities and they will need assistance from the international community.
3. Including up to date incidence and case mortality rate of the coronavirus infections based on the total population will help the general public better assess the impact and reduce overreacted social panic.
4. For public reporting, countries especially those with suspected and confirmed cases of the COVID-19 should actively report to the WHO for further collaborations. Further relevant technical supports are required to construct a visual reporting system, with data and information coming from government daily updates and social media.
5. It is important for other countries to timely share the indicators information with the WHO, and a global visual surveillance system is increasingly required, together with current modules in daily situation report, travel advice, technical guidance, and global research [9].

## Conclusion

Due to the sudden outbreak of the COVID-19, China’s health system is being verified for its effectiveness in responses. Although the outbreak has brought huge socioeconomic loss, the public health system for emergency events in China will be undoubtedly much improved in the near future. Moreover, public reporting has become an accumulative advantage for China to collaborate with the WHO and other countries. More research is needed to explore the influential factors behind the indicators for more efficient and more effective policy decisions and interventions.

## Data Availability

Not applicable.

## Abbreviations

2019-nCoV, or COVID-19, or SARS-CoV-2: Novel coronavirus
CDC: Center for Disease Control and Prevention
ICT: Information communications technology
WHO: World Health Organization
